# Beyond decriminalization: exploring the legalization of abortion globally

**DOI:** 10.3389/frph.2026.1771493

**Published:** 2026-02-10

**Authors:** Sanhita Ambast, Hazal Atay, Antonella Lavelanet

**Affiliations:** UNDP-UNFPA-UNICEF-WHO-World Bank Special Programme of Research, Development and Research Training in Human Reproduction (HRP), Department of Sexual, Maternal, Child, Adolescent Health and Ageing, World Health Organization, Geneva, Switzerland

**Keywords:** abortion, abortion decriminalization, human rights, legalization, reproductive health

## Abstract

Public health guidance and human rights standards recommend states to decriminalize abortion, and further recommend that they legalise abortion care to ensure that abortion seekers can access quality and human rights consistent care. This paper explores abortion decriminalization and legalization globally, including by clearly distinguishing between them to discuss the content, implications, and consequences of each. Furthermore, it uses data from the GAPD to identify trends and patterns in how countries have approached these regulatory questions, outlining four models of legalization identified through the texts of national laws and policies.

## 1 Introduction

The launch of the Global Abortion Policies Database (GAPD) has made it possible to systematically assess the legal and policy environments related to abortion across all WHO Member States, including tracking changes over time (1–3). Juxtaposing this country-level data with international human rights standards and public health guidance makes it possible to assess existing models of abortion regulation, and how these align with global norms. This has the potential to reveal emerging patterns in how countries approach the regulation of abortion, highlighting the models and strategies countries use.

The WHO guideline on abortion care outlines key legal and policy elements that support safe, accessible, and rights-based abortion services, distinguishing between the legalization and decriminalization of abortion (4). The Guideline recommends for the full decriminalization of abortion, meaning the removal of abortion from all penal or criminal laws, not applying other criminal offences to abortion (including offenses such as murder or manslaughter), eliminating any criminal penalties for obtaining, performing, assisting with, or sharing information about abortion (4). The Guideline notes that removing criminal penalties is only one step toward legalizing abortion. To ensure abortion services are not only lawful but also accessible, available, and high-quality, action must go beyond decriminalization, often requiring legal, policy, and other regulatory measures—such as medical codes, protocols, and clinical guidelines.

The WHO Abortion Care Guideline also contains specific recommendations relevant to the legalization of abortion (4), including recommendations against laws and other regulations that restrict abortion by grounds; and against laws and other regulations that prohibit abortion based on gestational age limits. The guideline further highlights the importance of an enabling environment to ensuring that people can access comprehensive and quality abortion care (4). This environment includes the respect for human rights including a supportive framework of law and policy, availability and accessibility of information, and a supportive, universally accessible, affordable and well-functioning health system. For states moving towards legalization, the guideline provides essential recommendations to ensure that laws, policies, and practice support quality abortion care.

Similarly, human rights bodies have asked states to move beyond the decriminalization of abortion (5). They have been clear that decriminalization of abortion is only part of, and distinct from, states' full obligation to ensure that abortion seekers can access quality and human rights consistent care. For example, they have recommended that states aim to ensure universal access to safe abortion care without discrimination for all individuals (6); remove existing barriers to safe and legal abortion (7); ensure access to medication for abortion (8); and ensure access to post abortion care irrespective of the legal status of abortion (6, 9).

In this context, there has been growing discussion in scholarship around what decriminalization and legalization entail, and how they differ from each other (10–12). This paper builds on that discourse by distinguishing between the processes of decriminalization and legalization. It uses data from the GAPD to identify trends in how countries have approached the regulatory question of decriminalization vs. legalization. Rather than providing a country-by-country analysis, this paper focuses on the broader patterns that emerge from this dataset. This approach allows us to highlight the diversity of policy models, without reducing the discussion to isolated national case studies.

## 2 Approaches to abortion decriminalization and legalization

### 2.1 The broader landscape of decriminalization and legalization

The criminalization and decriminalization of abortion can be understood as a spectrum. At one end is the full criminalisation of abortion, where abortion is either prohibited in all circumstances or there are no known legal grounds for abortion. At the other end, is full decriminalization of abortion, where individuals, including abortion seekers and providers face no criminal penalties. Most regulatory approaches fall somewhere in between these extremes, allowing abortion on certain grounds while imposing criminal penalties for abortions outside those permitted circumstances.

The legalization of abortion also exists on a spectrum, which runs alongside, but is distinct from, decriminalization. While decriminalization removes criminal penalties, legalization establishes affirmative rights and regulatory systems to ensure access. These processes are separate, and progress on one does not automatically lead to the other; jurisdictions can advance along each spectrum independently. At one end of this legalisation spectrum are frameworks that align more closely with global guidance, providing regulated pathways to services, consistent with human rights standards. At the other end are contexts where abortion is decriminalised in certain circumstances, but there are no legal or policy mechanisms to guarantee service provision or recognize abortion as a health entitlement. In such cases, there may be no criminal penalties for abortion; yet abortion is not fully legalized. Between these points lie partial frameworks, which may include regulations on how abortions can be accessed, which providers and facilities are authorized, permissible methods and medicines, and whether providers can refuse care on grounds of conscience.

### 2.2 Models of abortion legalization

**Figure 1:**
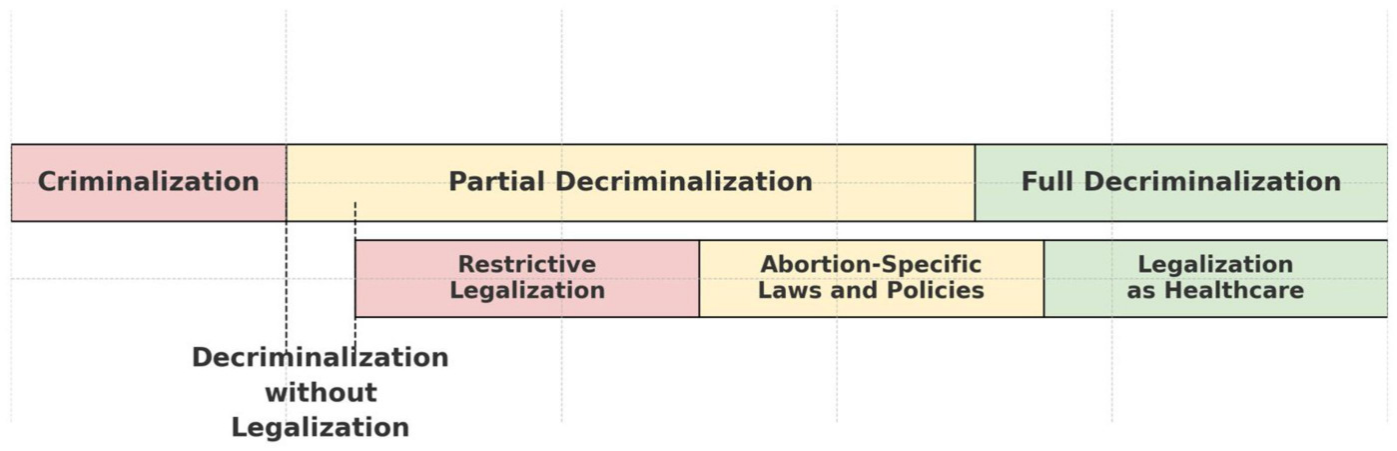
Decriminalization and legalization spectrums.

Countries have adopted a range of approaches to legalizing abortion. Based on our ongoing examination of the legal and policy sources compiled in the GAPD, this paper outlines four models, shown in Figure 1, that were identified through the analysis of national laws and policies. These models illustrate different approaches towards addressing key regulatory questions around abortion care, including the degree of criminalization, the level of legalization, and the types of legal sources relied upon. However, the GAPD does not capture how these laws and policies are implemented in practice; thus, these models do not speak to the quality of abortion services on the ground.

#### 2.2.1 Decriminalization without legalization

This regulatory model is characterized by the partial or complete removal of criminal penalties for abortion, without corresponding development of regulatory structures to enable access to care. While penalties for seeking and providing abortions may be removed in some circumstances, there is no framework that defines entitlement to abortion care, or addresses clinical and service delivery aspects, including pathways to access abortion. Guidance related to what methods of abortion are available, what pre and post abortion information provision should include, and other clinical and service delivery aspects are absent. In this approach, criminal law often remains the dominant source for abortion regulation in these countries.

#### 2.2.2 Restrictive model

This regulatory model is characterized by limited provisions for legalization and little guidance on how those provisions should be implemented. Abortion is usually decriminalised on limited grounds: with few and narrowly defined circumstances for lawful access. Criminal law generally remains the primary, and sometimes the sole, source for abortion regulation, describing legal grounds and gestational limits, and setting out who can perform an abortion, and where abortions may be performed.

#### 2.2.3 Regulation through abortion specific laws and policies

Among the regulatory patterns observed, one model positions abortion as a health intervention, governed by tailored legal and policy instruments, and yet abortion care is not embedded within general health care regulation. This approach often includes expanded grounds for access and, in some cases, abortion on request. It typically regulates issues such as provider qualifications, service locations, third-party consent, and conscientious objection through a combination of legislation, clinical guidelines, and ethics codes. In some jurisdictions, this distinctiveness is underscored by explicit recognition of a right to abortion in constitutional or statutory law.

#### 2.2.4 Abortion as health care

A fourth regulatory model integrates abortion care, either comprehensively or in specific component, into the general healthcare framework. In this approach, abortion provision is typically governed by general health care standards and criminal law is rarely used as a regulatory tool. Abortion is largely decriminalized, and many elements associated with legalization are structured to align with broader health care norms.

## 3 Discussion

This paper challenges the binary framing of abortion law reform as a simple move from prohibition to liberalization. Instead, it distinguishes between two related but distinct processes: decriminalization and legalization. Decriminalization of abortion is a key imperative, as the literature has shown that criminalization limits access to safe abortion and is likely to disproportionately impact marginalised groups (13). It can make health workers hesitant to provide abortions even when it is legal (13, 14), and can lead to a reduction in the number of trained abortion providers in the workforce (15, 16). Human rights law, international standards, and public health guidance consistently recommend the full decriminalization of abortion and emphasize the need to move beyond criminal frameworks altogether. Over time, trends show the gradual progress towards the decriminalization of abortion (17).

However, decriminalization alone is not sufficient. Where abortion is removed from criminal law but no regulatory framework for care exists, abortion seekers may not be enabled to access such care. Additionally, abortion providers may not know what they can provide within the limits of the law, impacting access to safer abortions. For these reasons, legalization of abortion care is crucial.

In this paper, we outline four framework models that demonstrate different approaches to key regulatory questions around abortion care. These models can influence how abortion care is implemented. For example, approaches to legalization relying on abortion-specific laws and policies, may signal that abortion requires a bespoke regulatory regime and heightened legal protections. Regulating abortion as an exception rather than as part of standard health care, including through restrictive models, can have health and human rights impacts. Such approaches can create additional barriers to access and care: they can delay access to care (18); introduce regulatory obstacles, such as the need for third party authorization (19); adversely impact the quality of medical education on abortion (20); and reinforce stigma (20, 21). Identifying this type of exceptionalism, both in medical practice and in the law, is a crucial first step towards creating an enabling environment for abortion care (4). Dwyer et al. have questioned the necessity of such laws (22), arguing that general health law, and related regulations, such as ethics codes and clinical protocols are often adequate to regulate abortion.

More generally, the multitude of approaches described in this paper reflects that there are different paths towards the legalization of abortion, which can depend on their historical, social, and legal contexts. While abortion law reform often involves sustained advocacy by feminists and activists (23–26), it can also be sudden and reactive (27, 28). For instance, a court may find that penalties for abortion violate constitutional and human rights guarantees, because of which it is decriminalised—or it can be the consequence of gradual and incremental policy and legal change (29, 30). While this paper does not explore historical drivers in detail, understanding these trajectories could shed light on how approaches to legalization evolve.

Legalizing abortion often requires reform across multiple legal domains, including criminal law, health law, administrative law, pharmaceutical regulation, professional regulation of health workers, and even employment and contract law (4). For example, eliminating unnecessary provider restrictions and allowing self-management of abortion could require reform in procurement regulation to ensure availability of medicines. It may also involve changes to pharmaceutical licensing and regulation to allow dispensing to abortion seekers, and criminal law to eliminate penalties tied to provider restrictions.

It is important to note that progress through the elements of legalization is rarely linear; jurisdictions may adopt different components of legalization at different points in time. One context might permit abortion on request up to a certain gestational limit and regulate providers and facilities yet omit regulation on conscientious objection by health care providers. Another might prohibit abortion on request but allow abortions on broad grounds with no gestational limit, while imposing third part authorization requirements.

Even where legal frameworks exist, the application of these laws and policies may also be limited by other barriers. These include limited knowledge among providers or abortion seekers (31), stigma, stereotypes, and personal biases (32, 33), lack of adequate medical equipment and staff (34, 35), and financial constraints that may impact individuals' ability to access abortion care (36). Addressing these challenges requires more than just legal reform; it requires accessibility of information, and a supportive, universally accessible, affordable and well-functioning health system (4). In this context, a crucial element of legalization becomes relevant: legalization implies that states are accountable to abortion seekers to ensure quality abortion care is available, accessible and affordable to all who seek it. This responsibility requires states to investigate and address all barriers to care, whether rooted in laws, policies, biases, or practice (5). Human rights standards and public health guidance, such as the Abortion care guideline, provide concrete and evidence-based steps that States can take to this end.

## 4 Conclusions

This paper contributes to the conversation on abortion decriminalization and legalization by distinguishing between these processes, in the context of public health and human rights guidance. The WHO Abortion care guideline acknowledges that the legalization and decriminalization of abortion are different and require different forms of action by States. Similarly, human rights bodies have asked States to move beyond decriminalization, towards the legalization of abortion care. Using data from the GAPD, we describe four approaches towards abortion regulation. Each approach reflects a different response to key regulatory questions including the degree of criminalization, level of legalization, and the nature of the sources used. This inquiry also raises important questions for the future around appropriate pathways to ensuring quality and accountable abortion care for all who seek it.

## Data Availability

Publicly available datasets were analyzed in this study. This data can be found here: Global Abortion Policies Database https://abortion-policies.srhr.org/.
